# Comprehensive analyses reveal the prognosis and biological function roles of chromatin regulators in lung adenocarcinoma

**DOI:** 10.18632/aging.204693

**Published:** 2023-05-05

**Authors:** Baishuang Yang, Xueyao Rong, Chen Jiang, Meihua Long, Aibin Liu, Qiong Chen

**Affiliations:** 1 National Clinical Research Center for Geriatric Disorders, Xiangya Hospital, Central South University, Changsha 410008, China; 2 Department of Geriatrics, Xiangya Hospital, Central South University, Changsha 410008, China; 3 Xiangya Lung Cancer Center, Xiangya Hospital, Central South University, Changsha 410008, China

**Keywords:** chromatin regulator, lung adenocarcinoma, prognostic, bioinformatic analyses, immune infiltration

## Abstract

The present study explored the prognosis and biological function roles of chromatin regulators (CRs) in patients with lung adenocarcinoma (LUAD). Using transcriptome profile and clinical follow-up data of LUAD dataset, we explored the molecular classification, developed, and validated a CR prognostic model, built an individual risk scoring system in LUAD, and compared the clinical and molecular characteristics between different subtypes and risk stratifications. We investigated the chemotherapy sensitivity and predicted potential immunotherapy response. Lastly, we collected the clinical samples and validated the prognosis and potential function role of NAPS2. Our study indicated that LUAD patients could be classified into two subtypes that had obviously different clinical background and molecular features. We constructed a prognostic model with eight CR genes, which was well validated in several other population cohort. We built high- and low-risk stratifications for LUAD patients. Patients from high-risk group were totally different from low-risk groups in clinical, biological function, gene mutation, microenvironment, and immune infiltration levels. We idented several potential molecular compounds for high-risk group treatment. We predicted that high-risk group may have poor immunotherapy response. We finally found that Neuronal PAS Domain Protein 2 (NPAS2) involved in the progression of LUAD via regulating cell adhesion. Our study indicated that CR involved in the progression of LUAD and affect their prognosis. Different therapeutic strategies should be developed for different molecular subtypes and risk stratifications. Our comprehensive analyses uncover specific determinants of CRs in LUAD and provides implications for investigating disease-associated CRs.

## INTRODUCTION

Lung cancer is not only one of the most common cancers throughout the world, but also the leading cause of death among cancer patients, with morbidity and mortality ranking first among all cancers around the world [1]. Adenocarcinoma of the lung (LUAD) is the most common subtype, representing the highest percentage of lung cancer [2]. For the treatment of lung cancer, in addition to surgery, radiotherapy, and chemotherapy, targeted therapy and immunotherapy provide new directions [3]. However, due to the problems of drug resistance, efficiency, and adverse reactions, its curative effects are still unsatisfactory.

Currently, the long-term survival outcome of lung cancer patients was less than satisfactory, and the five-year survival rate was from 4% to 17% [4]. Thus, the study of abnormally expressed genes in lung cancer, especially in lung adenocarcinoma, is of great importance to further elucidate the molecular mechanism of its development and progression, and to identify prognostic markers and therapy-related targets.

In eukaryotic cells, transcriptional regulation can occur at multiple levels, including RNA polymerases with different structure and function, corresponding broad-spectrum initiation factors, gene-specific regulators (DNA-binding proteins), and various coregulatory factors [5]. These coregulatory factors may contribute to the formation of transcription initiation complexes through chromatin modifications, such as histone acetylation and methylation, or more directly [6]. These regulation factors can be called as chromatin regulators (CRs). Over the past few years, studies have shown that epigenetic change, which is one of the most important tumor biomarkers, is caused by chromatin regulators (CR) [7]. CRs can dynamically regulate chromatin structure and epigenetic expression in response to endogenous and exogenous signals [8]. Somatic alterations and misexpression of CRs would reprogram the epigenetic profile of chromatin, leading to occurrences of common diseases, particularly cancers [9]. CR is an indispensable regulatory element in epigenetics [10]. Based on their roles in epigenetics, CR falls into three main categories: DNA methylators, histone modifiers, and chromatin remodeling factors. But these three categories are closely related to each other when it comes to biological processes [11]. Further studies have shown that aberrant expression of CRs correlated with many biological processes, including autophagy, proliferation apoptosis, and inflammation, indicating that CRs dysregulation may lead to the development of many diseases, including cancer [12–15].

In this study, we first explored the molecular subtypes based on CR genes and analyzed their clinical and molecular characteristics. Next, we established and validated a CR prognostic prediction model in LUAD patients, and built a nomogram scoring system for assessing individual’s prognosis risk. Then, we made comparisons for clinical characteristics, gene alteration and mutation, biological function, tumor microenvironment and immune infiltration levels for different risk stratifications. After that, we investigated the chemotherapy sensitivity and predicted potential immunotherapy response. Lastly, we collected the clinical samples and validated the prognosis and potential function role of NAPS2 (a key molecular of cell adhesion) in LUAD. Our study provided new insights for the function role of CR in LUAD.

## MATERIALS AND METHODS

### Patients and samples

This study included data was from public data platform (The Cancer Genome Atlas, https://portal.gdc.cancer.gov/), including 58 normal samples and 517 LUAD samples. The clinical characteristics and follow-up information were used. Based on median absolute deviation (MAD)>0.5 and follow-up data, we excluded some samples. 870 CR genes were obtained from previous study [16].

A total of 429 formalin-fixed paraffin-embedded lung samples, including 41 benign lung disease tissues and 388 LUAD tissues, were obtained during surgery or needed biopsy. The clinical and follow-up information were also achieved. These samples were from Jiangmen Central Hospital (Guangdong, China) between January 2010 and December 2020.

### Molecular subtypes

The consensus clustering was used for molecular subtyping, which was carried out using ‘ConsensusClusterPlus’ R packages. The optimal number was determined by the visualization of the consensus matrix [17]. Principal Component Analysis (PCA) and Distributed Stochastic Neighborhood Integration (tSNE) were used to confirm the distribution of molecular sub-types [18]. Gene set variation analysis (GSVA) was adopted to compare the pathway enrichment difference between two molecular subtypes [19]. The CR genes expression profile and clinical characteristics were also compared.

### Development and validation of the prognostic model

We developed a CR prognostic model using CR genes according to the following steps: we identified the prognosis-related CR genes using the univariate cox regression. The least absolute shrinkage and selection operator (LASSO) method was performed to identify the optimal gene groups from the prognosis-related CR genes, and the regression coefficients (β) were also calculated. We then calculated the risk score using the following formula: risk score=β_1_*Gene expression_1_+…+β_N_*Gene expression_(N)_. The sample data was divided into high-risk and low-risk groups according to the median of risk score. Kaplan-Meier analysis was conducted to compare survival outcomes across high- and low-risk groups. PCA was used for visualizing the risk distribution. Using the calculation formula of risk score, we obtained the risk score of each sample in the GEO validation dataset (GSE31210, GSE37445, GSE50081, GSE19188, GSE30219) and ArrayExpress validation dataset (E-MTAB-923). Similarly, we also compared the survival curve of high- and low-risk groups in the two validation datasets.

### Establishment and assessment of individual risk scoring system

To investigate the independent prognosis correlation of the risk score, we performed the univariate and multivariate cox regression analysis with hazard ratios (HRs) and corresponding 95% confidence intervals (CIs). We built a visual nomogram scoring system to assess the induvial risk score based on the risk stratification and clinical information. The predictive ability was assessed using the receiving operating characteristics curve (ROC) and decisive curve. The model fit was assessed using the calibration curve that showed the correlation of actual outcomes with nomogram-predicted outcomes.

### GO and KEGG pathway enrichment and gene alteration analysis

Based on risk stratification, we obtained the differentially expressed genes (DGEs) between high- and low-risk groups. Then, Gene Ontology and KEGG pathway analyses were performed using the “clusterProfiler” package. Gene alterations, variant classifications and types, and co-occurrence and mutually exclusive were compared between two risk groups using “limma” R package.

### Tumor microenvironment and immune infiltration analysis

We calculated the ESTIMAT, stromal and immune scores to evaluate the tumor microenvironment of differ groups. The immune infiltration level was evaluated using the CIBERSORT method, including 22 kinds of immune cells and function, which was used in previous study [20].

### Chemotherapy sensitivity and immune response

The chemotherapy sensitivity was assessed via IC50 of compounds of different risk groups. The elevated IC50 means chemotherapy resistant. This process was finished using ‘pRRophetic’ R package. Using pre-treatment expression profiles of tumor patients, we estimated Tumor Immune Dysfunction and Exclusion (TIDE) to predict patient’s immunotherapy response, which was achieved in web application of TIDE (http://tide.dfci.harvard.edu/). The elevated TIDE levels mean good immunotherapy response.

### Immunohistochemistry

We detected the NPAS2, integrin beta4 and p-FAK expression in 388 LUAD samples using the immunohistochemistry. The process was previously described [21]. The specific steps for immunohistochemistry can be found in the Supplementary Material 1.

### Statistical analysis

For variables normal distributed variables, data are presented as the mean ± standard deviation (SD) and the statistical difference between the two groups was determined using the student’s t-test. The statistical differences between multiple groups were determined by One-way ANOVA. Nonparametric test (Mann-Whitney U-test) was used to assess the significance of the differences. Survival curves for patients were utilized by the Kaplan-Meier method. P < 0.05 indicated a statistically significant difference. All analyses were conducted by SPSS 23.0 software (IBM Corp, Chicago, IL, USA) and diagrams were produced by GraphPad Prism 8.0 software (GraphPad Inc, San Diego, CA, USA).

### Data availability

The data can be partly available from the public database. Data from experiments can be available from the corresponding authors upon request.

## RESULTS

### Molecular subtypes based on chromatin regulators

**Figure 1:**
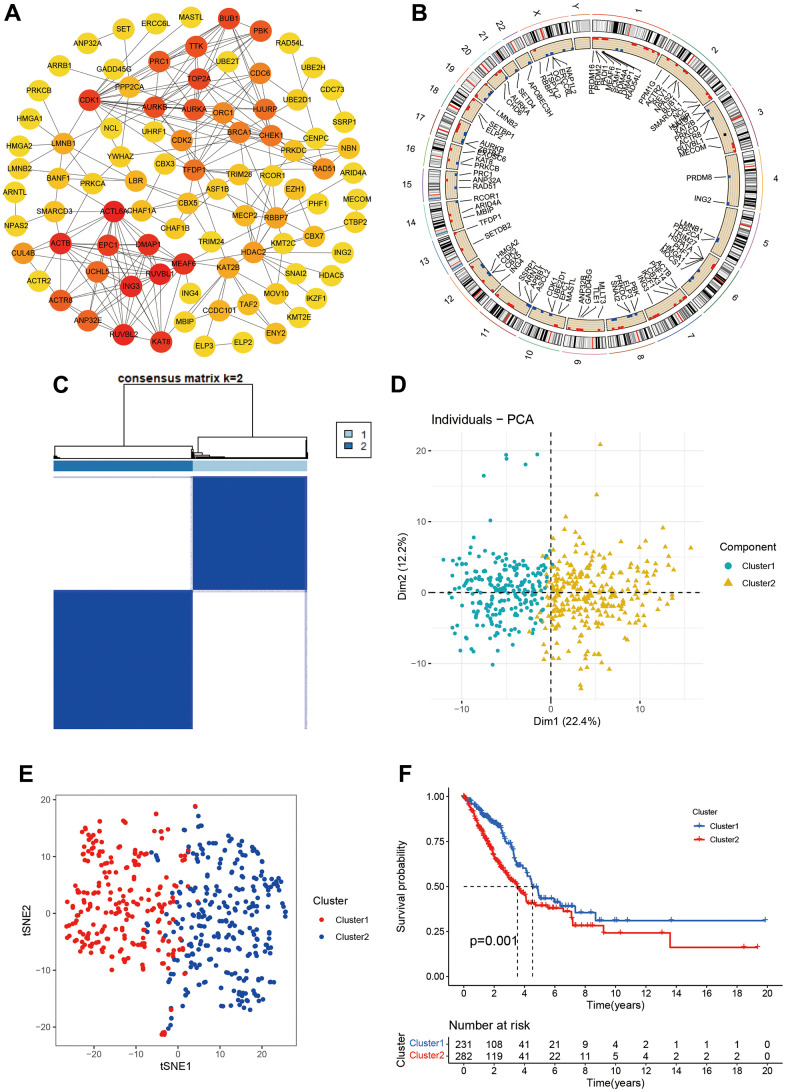
**Molecular subtypes based on chromatin regulator.** (**A**) Protein-protein interaction of chromatin regulators; (**B**) The position of CRs on the chromosome; (**C**) Consensus matrix indented two subtypes. (**D**, **E**) PCA and corrected PCA identified two components. (**F**) Kaplan-Merrie survival curves of cluster 1 and cluster 2.

We obtained 870 CR genes from previous publications. After screening using Median absolute deviation (MAD) >0.5 and univariate cox regression (P<0.05), we identified 153 CR genes for molecular subtypes. We firstly built the PPI network for 153 CR genes and we found ACTL6A, RUVBL1, RUVBL2, ING3, ACTB, DMAP1, MEAF6, KAT8, EPC1, and CDK1 top 10 hub genes (Figure 1A). Then, we explored the location information and mutation frequency of these CR genes (Figure 1B). Using ConsensusClusterPlus package, we explored the molecular subtypes and found LUAD can be sharply and clearly divided into two clusters when the optimal k value is 2 (cluster 1 and cluster 2, Figure 1C). The PCA and t-distributed stochastic neighbor embedding method also showed a two-dimensional distribution (Figure 1D, 1E). Finally, we performed the Kaplan-Meier analysis and found the cluster 2 had poorer prognosis than cluster 1 (Figure 1F). These results suggested LUAD patients could be separated into two subtypes based on CR genes.

**Figure 2:**
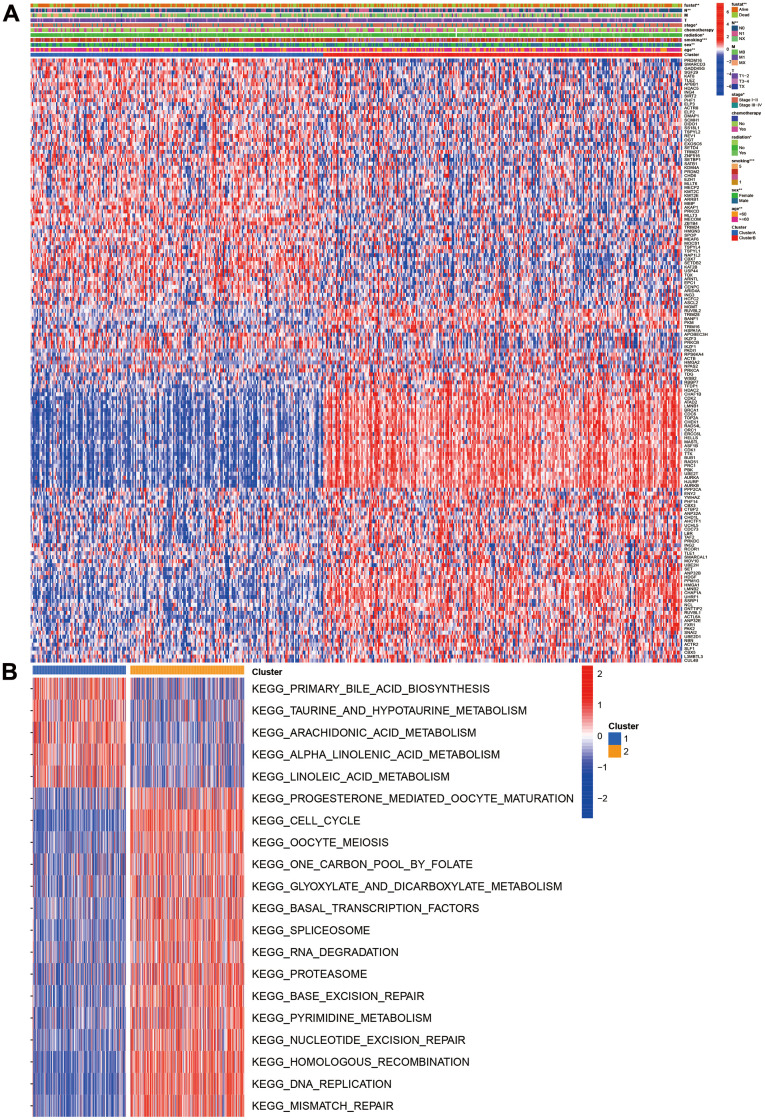
**Clinical and function characteristics between two clusters.** (**A**) Correlations of clinical characteristics with molecular subtypes; (**B**) Gene set variation analysis of two clusters.

We also investigated the CR expression levels and compared the clinical characteristics of two clusters. Compared with cluster 1, the cluster 2 had higher ratios of male, smoking, no radiation, pathology stage, N stage, and poor prognosis. ATAD2, LMNB1, BRCA1, CDC6, TOP2A, CHEK1, RAD54L, ORC1, ERCC6L, MASTL, ASF1B, CDK1, TTK, BUB1, RAD51, PRC1, PBK, and UBE2T were markedly increased in the cluster 2 (Figure 2A). The GSVA results suggested that some pathways were positively enriched in the cluster 2, including cell cycle, oocyte meiosis, one carbon pool by folate, glyoxylate and dicarboxylate metabolism, RNA degradation, pyrimidine metabolism, homologous recombination, DNA replication, mismatch repair, and proteasome. The cluster 1 had higher primary bile acid biosynthesis, taurine and hypo taurine metabolism, arachidonic and linoleic acid metabolism (Figure 2B).

### Development and validation of prognostic model based on chromatin regulators

**Figure 3:**
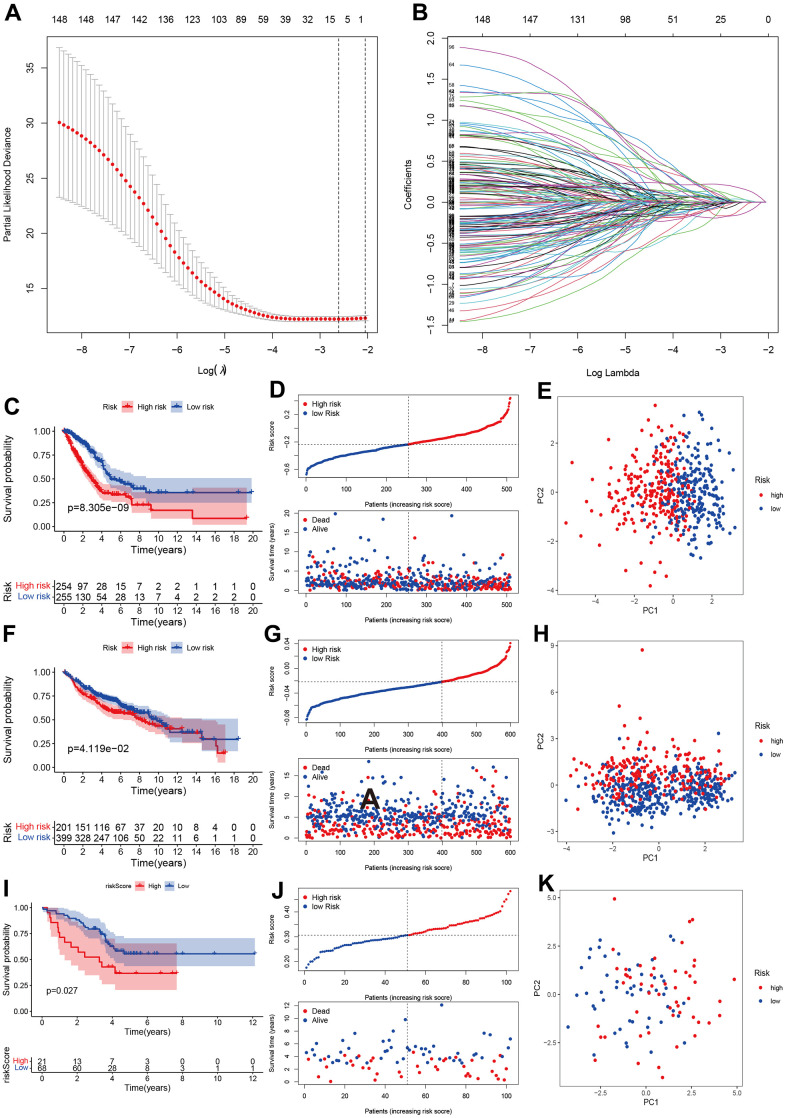
**Development and validation of prognostics model based on CRs.** (**A**, **B**) LASSO regression identified optimal gene number in the model. (**C**–**E**) Kaplan-Merri survival curve, risk score and risk components in TCGA training group. (**F**–**H**) Kaplan-Merri survival curve, risk score (best cut-off value) and risk components in GEO validation dataset (GSE31210, GSE37445, GSE50081, GSE19188, GSE30219); (**I**–**K**) Kaplan-Merri survival curve, risk score and risk components in ArrayExpress validation dataset (E-MTAB-923).

We developed a prognostic model for LUAD patients based on CR genes. We first carried out the LASSO regression and achieved the number of genes in the model. Eight genes entered final model (SETDB2, NPAS2, HMGA2, TLE1, HJURP, SNAI2, PHF1, PRKCD; Figure 3A, 3B). We then calculated the risk score of each sample using the regression coefficient multiplied by gene expression. All patients were divided into two groups (high- and low-risk groups) referring to the median of risk score. The Kaplan-Meier analysis indicated that high-risk group had poorer OS than the low-risk group (Figure 3C, 3D). PCA showed two obvious risk groups distributions (Figure 3E). We further validated the prognostic model in two datasets including GEO validation dataset (GSE31210, GSE37445, GSE50081, GSE19188, GSE30219) and ArrayExpress dataset (E-MTAB-923). Similarly, the prognosis of high-risk group was still poorer than the low-risk group (Figure 3F, 3G), and PCA showed two-dimensional data distributions (Figure 3H). Furthermore, another cohort data also confirmed our model (Figure 3I–3K).

### Establishment and assessment of individual risk scoring system

**Figure 4:**
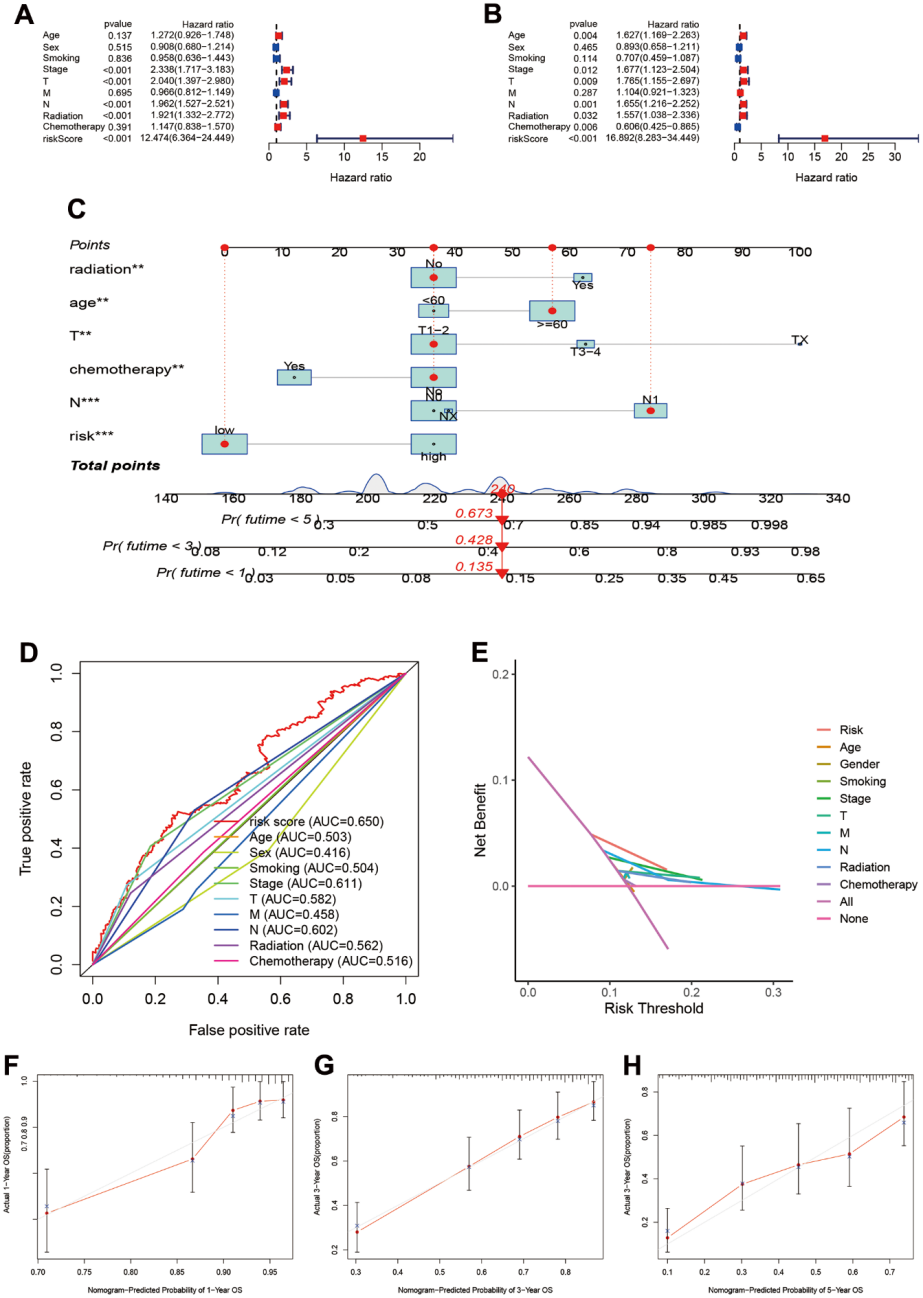
**Establishment and assessment of individual risk scoring system.** (**A**, **B**) Univariate and multivariate cox regression for risk score in LUAD; (**C**) Nomogram plot for individual risk assessment. (**D**) Comparisons of predictive ability between risk score and other clinical parameters; (**E**) Decision curve analysis for survival risk nomogram; (**F**–**H**) Calibration plots for 1-year, 3-year and 5-year OS.

The univariate cox regression showed that risk score, pathology stage, T, N stage, and radiation were associated with prognosis (Figure 4A). The multivariate cox regression suggested that risk score can independently predict the prognosis in LUAD patients (HR=16.892, 95%CI:8.283-34.449, P<0.001; Figure 4B). Age, pathology Stage, T and N stage, radiation and chemotherapy were also associated with prognosis in LUAD.

To assess prognosis of the individual, we built individual risk scoring system using the nomogram. This scoring system included the risk score and clinical characteristics. Our results indicated the 1-year, 3-year and 5-year survival rate were 0.673, 0.428, and 0.135 (Figure 4C). The ROC showed the risk score achieved the highest predictive ability for prognosis compared to other clinical parameters (AUC=0.650, Figure 4D). The deceive curve indicated that risk score had best net benefit when the risk threshold was 20% (Figure 4E). Meanwhile, we used the calibration curve to assess the predictive ability of model. The predictive outcomes and true data had good fits (Figure 4F–4H).

### Function and pathway enrichment and gene alterations in different risk stratification

**Figure 5:**
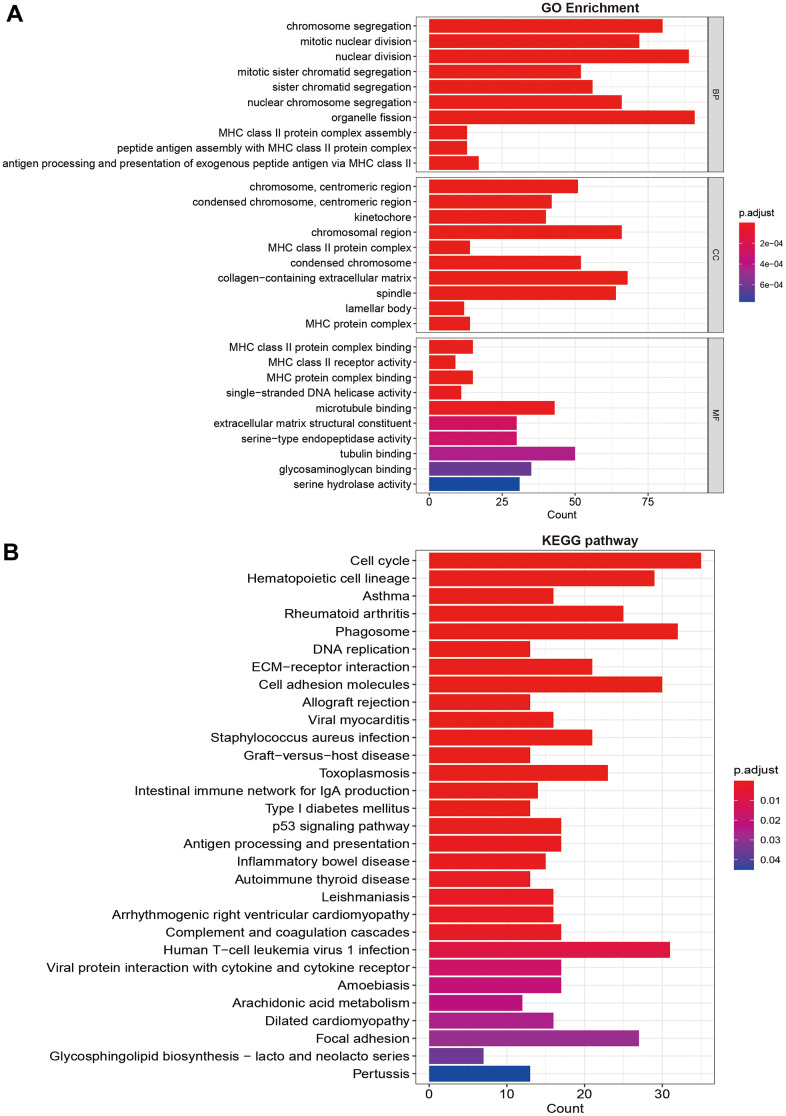
**Functional and pathway enrichment analysis.** (**A**) GO function enrichment; (**B**) KEGG pathway analysis.

Through expression differences analysis, we obtained 371 differentially expression genes from the results. The GO function enrichment suggested that high-risk group was mainly enriched in chromosome segregation, nuclear division, MHC class II protein complex assembly, single-stranded DNA helicase activity (Figure 5A). The pathway analysis showed that high-risk group mainly enriched in cell cycle, DNA replication, ECM-receptor interaction, cell adhesion molecules, p53 signaling pathway, and focal adhesion (Figure 5B).

**Figure 6:**
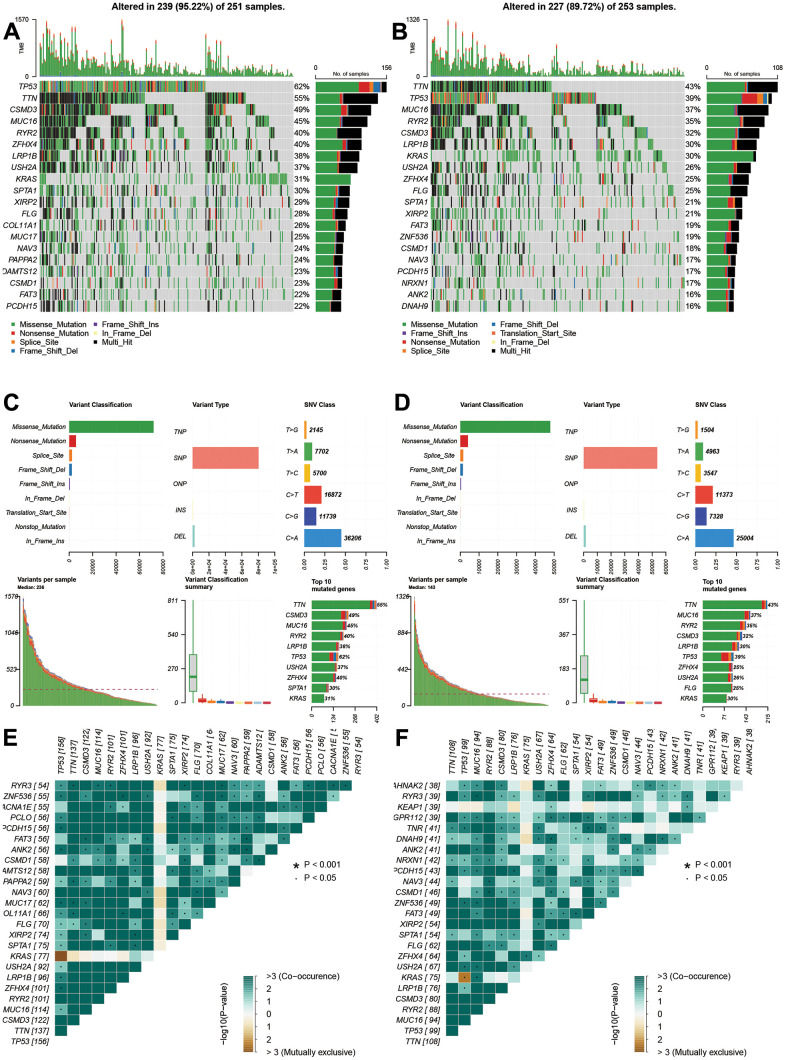
**Gene alterations and variations of different risk groups.** (**A**, **B**) Genes alterations of high- and low-risk groups. (**C**, **D**) Variant classification and types of high- and low-risk groups. (**E**, **F**) Co-occurrences and mutually exclusive status of high- and low-risk groups.

The gene alterations were also analyzed. The high-risk group had higher alteration levels than low-risk groups. The top 10 gene alterations were TP53, CSMD3, TTN, ZFHX4, USH2A, MUC16, RYR2, KRAS, LRP1B, and SPTA1 for high-risk group (Figure 6A), and the top 10 genes were TTN, TP53, MUC16, RYR2, CSMD3, LRP1B, KRAS, USH2A, ZFHX4, and FLG for low-risk group (Figure 6B). No significant differences were observed in variant classification and variant type, and SNV class for two risk group (Figure 6C, 6D). For high-frequency gene alteration, the co-occurrence showed similar trends between high-risk and low-risk groups. Top altered genes were highly correlated (Figure 6E, 6F) for two risk groups. There are still some differences for two groups such as MUC16, KRAS, LRP1B, USH2A, and ZFHX4.

### Correlations of different risk stratification with immune status

**Figure 7:**
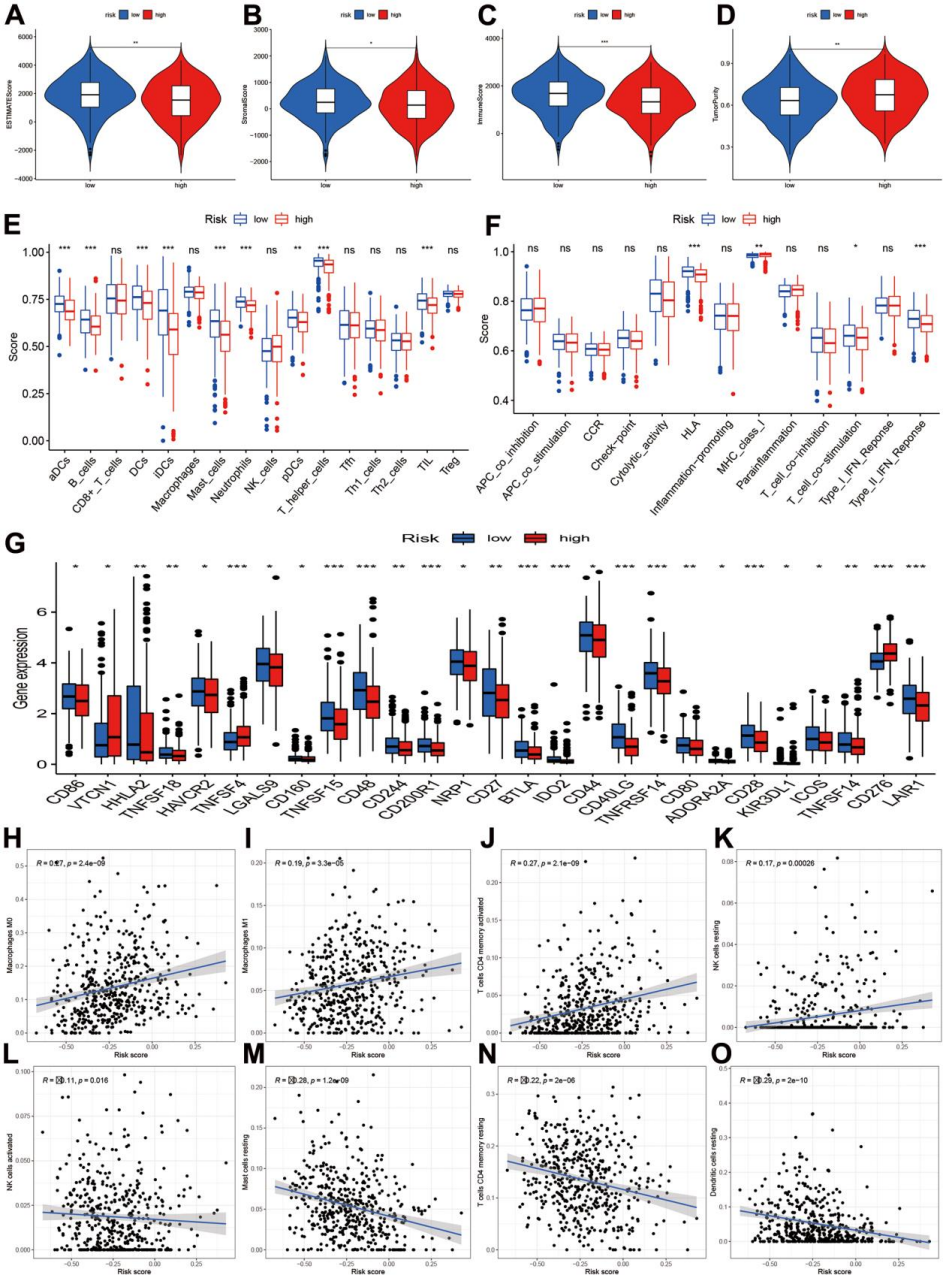
**Tumor microenvironment and immune infiltration level.** (**A**–**D**) Comparisons of ESTIMATE, stromal, immune and tumor purity between high- and low-risk groups; (**E**, **F**) Immune cell infiltration and function score between high- and low-risk group. (**G**) Comparisons of immune-related genes expression levels between two risk groups. (**H**–**O**) Scatter plot for the correlations of risk score with some immune infiltration cells, including macrophages M0, M1, T cells CD4 memory, NK cells, Mast cells, Dendritic cells.

The tumor microenvironment and immune infiltration levels were also evaluated. The results showed the low-risk had higher ESTIMATE, stromal and immune score (Figure 7A–7C, *P*<0.05). However, the tumor purity increased in the low-risk group (Figure 7D, *P*<0.01). The infiltrations levels of high-risk group were lower than low-risk group, including aDCs, B cells, DCs, iDCs, mast cells, neutrophils, NK cells, pDCs, T helper cells, TIL, HLA, and type II IFN response (Figure 7E). However, the MHC class I was higher in the high-risk groups. We further compared the immune-checked point genes. The VTCN1, TNFSF4, CD276 genes presented elevated levels in the high-risk groups (Figure 7G). Furthermore, macrophages M0, M1, T cells CD4 memory activated, and NK cells resting were positively related to risk score (Figure 7H–7K), while NK cells activated, mast cell resting, T cells CD4 memory resting, and dendritic cells resting were negatively related to risk score (Figure 7L–7O).

### Chemotherapy sensitivity and immune response assessment

**Figure 8:**
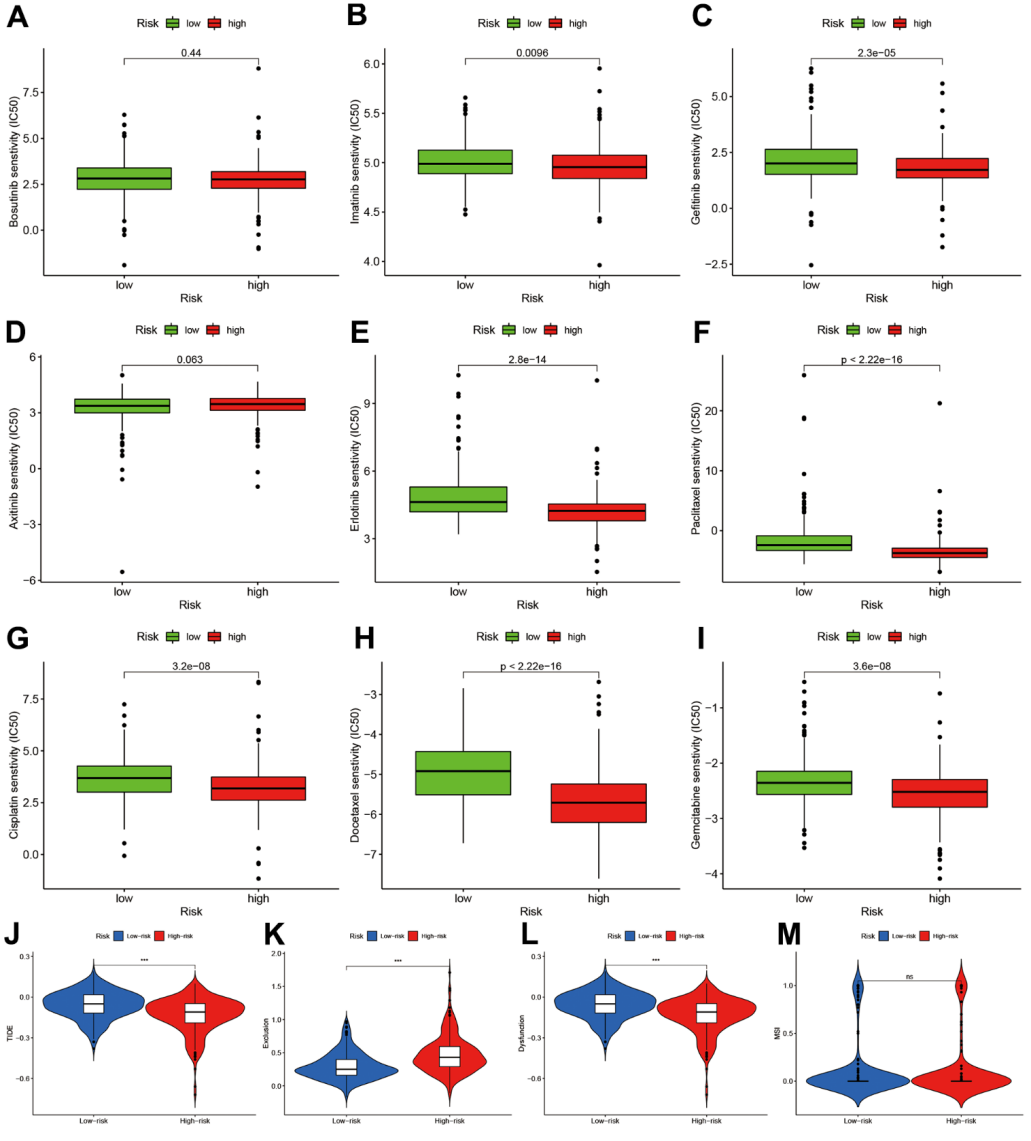
**Chemotherapy sensitivity and immunotherapy response assessment.** (**A**–**I**) IC50 of nine kinds of chemotherapy drug between high- and low-risk groups. (**J**–**M**) Immunotherapy response prediction based on Tumor Immune Dysfunction and Exclude module.

To explore the potential treatment molecular, we compared the IC50 of two groups in some compounds. The results suggested that Imatinib and Gefitinib may be sensitivity in high-risk group (Figure 8A–8C). Erlotinib, Paclitaxel, Cisplatin, Docetaxel, and Gemcitabine may show chemotherapy resistance for high-risk group (Figure 8D–8I).

We also predicted the immunotherapy responses based on TIDE estimation. The high-risk group had lower TIDE levels (Figure 8J), and the exclusion and dysfunction were elevated (Figure 8K, 8L). There was no difference in MSI between two groups.

### Prognosis and function roles of NPAS2 in LUAD

**Figure 9:**
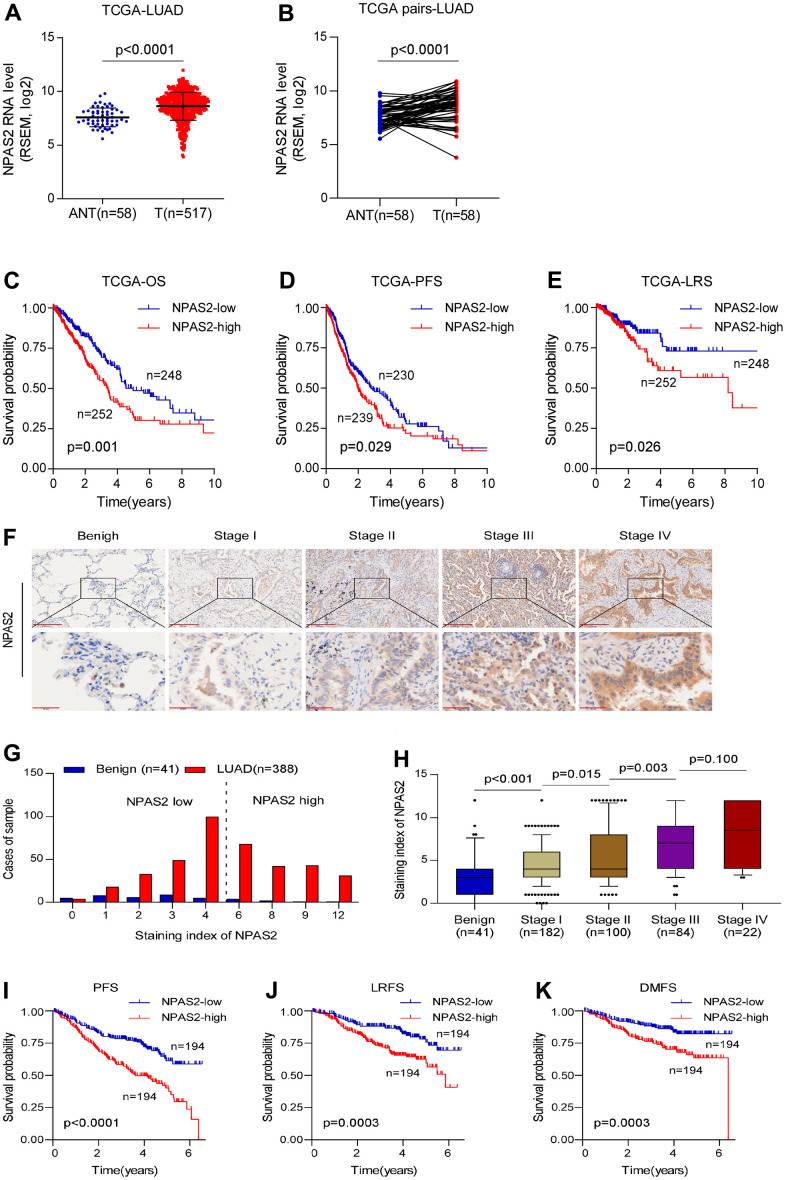
**NPAS2 was associated with prognosis in LUAD.** (**A**, **B**) NPAS2 expression levels between tumor and normal sample in TCGA; (**C**–**E**) High-expressed NPAS2 was associated with poor OS, PFS and LRS in LUAD based on TCGA; (**F**–**H**) NPAS2 expression increased with advanced stage. (**I**–**K**) High-expressed NPAS2 was associated with poor PFS, LRS and DMFS of LUAD patients in an independent cohort.

The reason we chose NPAS2 for validation is that NPAS2 was only significantly elevated in tumor but associated with poor prognosis. We collected 388 samples of LUAD patients to validate our findings. The clinical information of 388 patients and normal tissues were presented in Supplementary Tables 1, 2. We firstly analyzed the NPAS2 expression in TCGA. We found significance differences between benign and tumor samples (Figure 9A, 9B). The Kaplan-Meier curve indicated that high-expressed NPAS2 was associated with poor OS, PFS and LRS (Figure 9C–9E). We next detected the expression of NPAS2 in 388 LUAD samples using immunohistochemical method. The NPAS2 expressed increased with elevated stage (Figure 9F–9H and Supplementary Table 3). The univariate and multivariate cox regression also indicated that high-expressed NPAS2 was an independent prognosis factor (Supplementary Tables 4, 5). We further confirmed that high-expressed NPAS2 was related to poor PFS, LRFS and DMFS in LUAD (Figure 9I–9K).

**Figure 10:**
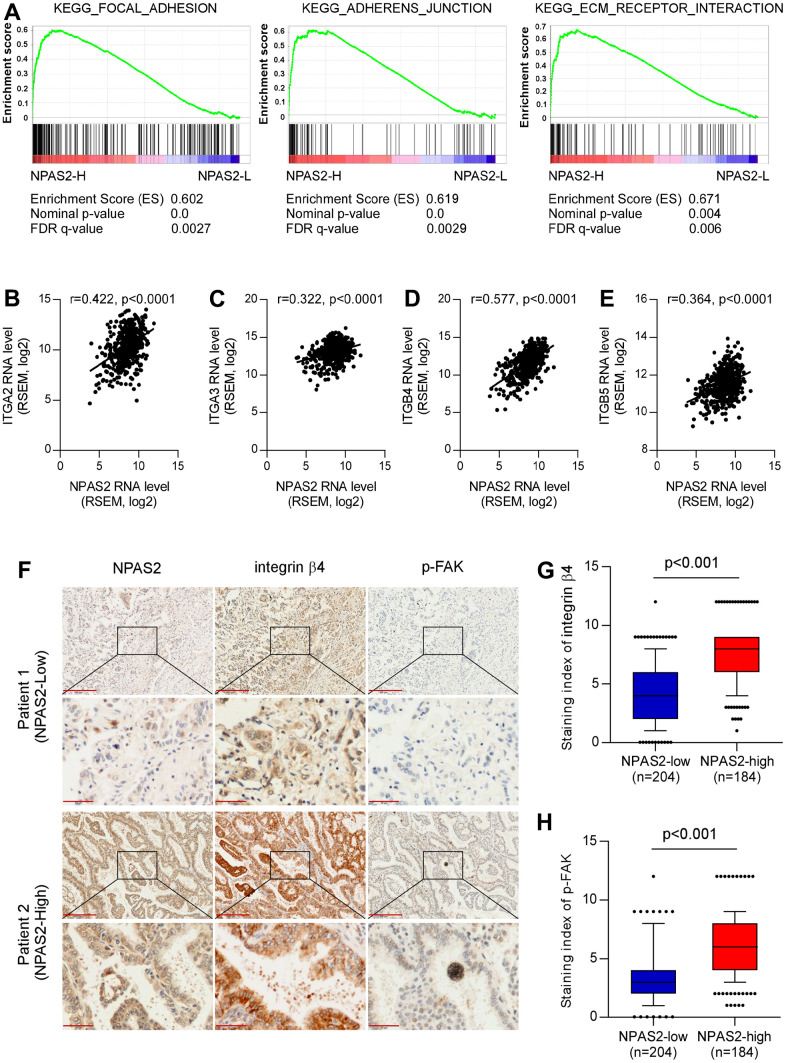
**Potential mechanisms of NPAS2 in the progression of LUAD.** (**A**) KEGG pathways enrichment of NPAS2. (**B**–**E**) Correlations of NPAS2 with ITGA2, ITGA3. ITGB4, and ITGB5; (**F**) Immunohistochemical showed the correlations of NPAS2 with integrin beta4 and p-FAK; (**G**) NPAS2-high group had elevated integrin beta 4 levels; (**H**) NPAS2-high group had elevated p-FAK levels.

The KEGG pathway analysis was performed, and the NPAS2 was positively enriched in focal adhesion, adherent’s junction, and ECM receptor interaction (Figure 10A). We also found NPAS2 was positively associated with ITGA2, ITGA3, ITGB4, and ITGB5 (Figure 9B–9E), which belong to integrin family that was closely to cell adhesion [22, 23].

We further explored the association between NPAS2 and integrin beta4 and p-FAK that were key molecular of cell adhesion. The immunohistochemical tests indicated that the integrin beta 4 and p-FAK were significantly elevated in high-expressed NPAS2 group. We speculated that NPAS2 involved in the progression of LUAD via regulating cell adhesion.

## DISCUSSION

In the present study, we found that: (1) LUAD can be divided into two molecular subtypes based on CR gene expression profile, and distinctly different clinical characteristics and molecular biological features were found for two molecular subtypes. (2) Using eight CR genes, we developed and validated a prognostic model in LUAD patients. This model was well validated in several independent cohort data. The risk score was an independent prognosis predictive factor for LUAD patients. (3) The nomogram scoring system was established for individual risk prediction. (4) Risk stratification based on prognostic model can distinguish high-risk patients from clinical characteristics, biological function, gene alterations and mutation, immune infiltration background. (5) Risk stratification also affect the chemotherapy sensitivity and immunotherapy response. (6) Using the detection of clinical samples, we found NNPAS2 involved in the progression of LUAD via regulating cell adhesion. Our study provided new insights for the functional role of CR in LUAD.

Previous studies had divided lung cancer into many molecular subtypes. However, these subtypes still cannot fully include all kinds of lung cancers and their clinical characteristics due to high heterogeneity of tumor [24]. In our study, we found LUAD can be separated into two molecular subtypes with different prognosis based on CR genes expression profile. The molecular features and pathway enrichment of two molecular subtypes are markedly different. Our results provided new perspectives for LUAD prognosis management. A recent study also explored the function of CR in LUAD [25]. But there were still some obviously different between our study and previous. First, the method of prognostic establishment was different. Previous study included model genes via multivariate cox regression. Our study identified the model’s genes for prognosis-related genes confirmed by univariate cox regression via LASSO regression. Our algorithm had many advantages. Compared with multivariate cox regression, LASSO can more effectively avoid multicollinearity of high-latitude data. It can also control the complexity of the model through a series of parameters to avoid over-fitting [26]. That’s why previous model included two genes that were not significant in the multivariate cox regression. Second, both our study and previous identified that risk score was an independent prognosis factor in LUAD. However, our study adjusted for more confounding factor including smoking, radiation and chemotherapy that were not included in previous studies. Thirdly, we not only developed and validated a prognostic model but also established an individual risk scoring system using nomogram, which was not presented in previous study. Finally, we collected 388 clinical samples and validated the prognosis and function role of NPAS2 in LUAD, and previous study only detected the expression of several CR genes in cell lines. Besides, we also evaluated the chemotherapy sensitivity and predicted immunotherapy response that were not done in previous study. Anyway, for such a study topic, our study was different from previous study in many places.

Previous studies also built some other prognosis model. Wang developed a 16 cuproptosis-related lncRNA model in LUAD, and the AUC was more than 0.8 [27]. Nguyen built a lepidic gene signature that predicts patient prognosis, and the AUC was 0.744 [28]. Though these two modes have higher AUC but they also more genes in the model, which was inconvenient for predicting in practice. For predicting model, it should be easily and inexpensive used. Gong also built a pyroptosis-related prognosis model, and the largest AUC was 0.677 that was close to our results [29]. The future study should be combined more gene sets to obtain high predictive ability.

Immunotherapy of lung cancer is a new therapeutic method, and some cancer patient’s cancer have achieved satisfactory therapeutic effect [30]. We analyzed the immune infiltration levels of different risk stratification. The infiltrations levels of high-risk group were lower than low-risk groups in aDCs, B cells, DCs, iDCs, mast cells, neutrophils, NK cells, pDCs, T helper cells, TIL, HLA, and type II IFN response. However, the MHC class I was higher in the high-risk groups. We further compared the immune-checked point genes between high- and low-risk groups. The VTCN1, TNFSF4, CD276 genes presented elevated levels in the high-risk groups. These results indicated that CR risk stratification can affect the immune infiltration. To explore whether immunotherapy was affected or not, we predicted the immunotherapy based on TIDE. We found high-risk group had decreased TIDE levels, which means that immune checkpoint blocking therapy (ICB) has poor efficacy and short survival after immunotherapy treatment [31].

GO enrichment indicated that high-risk group were chromosome segregation, nuclear division, MHC class II protein complex assembly, single-stranded DNA helicase activity. The KEGG pathway analysis showed that high-risk group was mainly enriched in cell cycle, DNA replication, ECM-receptor interaction, cell adhesion molecules, p53 signaling pathway, and focal adhesion. Both of two results indicated that cell adhesion may play an important role in the progression of LUAD. Cell adhesion was closely associated with invasion and metastasis of lung cancer [32–34]. As we all know, lung cancer is highly susceptible to metastasis. For the NPAS2 validation results, we found that high-expressed NPAS2 is positivity enriched in focal adhesion, adherens junction and ECM receptor interaction, which are important signal pathways in lung cancer metastasis [35–37]. We also found that NPAS2 were associated with integrin molecular including ITGA2, ITGA3, ITGB4, and ITGB5. Integrins mediate the reaction between cells and cells and between cells and matrix, and participate in various physiological processes such as cell signal transmission, cell adhesion and migration, control of cell differentiation, proliferation, and regulation, among which cell adhesion and signal transmission are two basic functions [38]. The invasion and metastasis of lung cancer is the result of a series of interacted steps, involving lung cancer cell adhesion, signal transmission, angiogenesis, apoptosis and other links, and integrin is closely related to these links [39–42]. The clinical sample analyses indicated that high-expressed NPAS2 group had elevated integrin beta4 and p-FAK that were key molecular of cell adhesion [43, 44]. These results supported that CR genes including NPAS2 involved in the progression of LUAD via regulating cell adhesion.

Our study had several limitations. One of them was that molecular subtypes were complex process and single gene set had some restrictions in tumor clustering, and multiple gene set should be included. But our results still provide some clues for further research. The other is that our validation results were performed in clinical samples and biological function were still confirmed via cell experiments.

In conclusion, our study indicated that CR genes involved in the progression of LUAD and affect their prognosis. Different therapeutic strategies should be developed for different molecular subtypes and risk stratifications. Our integrative analysis reveals specific determinants of CRs in LUAD and provides implications for investigating disease-associated CRs.

## Supplementary Material

Supplementary Material 1

Supplementary Tables
