# Supplementary material for: Comprehensive analyses reveal the prognosis and biological function roles of chromatin regulators in lung adenocarcinoma
Source: Aging (Albany NY). 2023 May 5;15(9):3598–620. doi: 10.18632/aging.204693 (PMC10449281; doi:10.18632/aging.204693)
Supplement: Supplementary Material 1 [file aging-15-204693-s001.pdf]

SUPPLEMENTARY MATERIALS

Supplementary Material 1

The specific steps for immunohistochemistry

The sides of a formalin-fixed paraffin-embedded section were antigen retrieved in Tris/EDTA (TE; pH 9.0) buffer, 10 minutes by microwave heating, blocked by hydrogen peroxide and goat serum, respectively, incubated overnight at 4° C in a humidified chamber with anti-NPAS2 antibody ((Invitrogen, Carlsbad, CA, USA), anti-integrin β4 antibody (Cell Signaling Technology, Danvers, MA, USA) and anti-p-FAK antibody (Cell Signaling Technology, Danvers, MA, USA) diluted in Antibody Diluent (Abcam, Cambridge, MA, USA), respectively. After incubation, slides were washed in Tris-buffered saline (TBS)/0.05% Tween 20, incubated with biotin-conjugated secondary antibody (Proteintech, Wuhan, China) and peroxidase-conjugated streptavidin (Proteintech), 30 min at 37° C, respectively, stained by the 3,30-diaminobenzidine (DAB) Enhanced Liquid Substrate System (Sigma-Aldrich, St. Louis, MO, USA). We quantified the NPAS2, integrin beta4 and p-FAK using staining index (SI). The sample was scored according to the following criteria: 0 for no positive tumor cells, 1 for 0-10%, 2 for 10-35%, 3 for 36-70%, and 4 for more than 70% positive tumor cells. Staining intensity was graded according to the following criteria: 0 (no staining), 1 (weak staining, light yellow), 2 (moderate staining, yellow brown), and 3 (strong staining, brown). SI was calculated as the product of staining intensity score and the proportion of positive tumor cells. Images were collected under 10× and 40× objective magnification in human lung tissues using M8 Digital Microscopy (PreciPoint, Freising, Bavaria, Germany).
