# Supplementary material for: Comprehensive analyses reveal the prognosis and biological function roles of chromatin regulators in lung adenocarcinoma
Source: Aging (Albany NY). 2023 May 5;15(9):3598–620. doi: 10.18632/aging.204693 (PMC10449281; doi:10.18632/aging.204693)
Supplement: Supplementary Tables [file aging-15-204693-s002.pdf]

## SUPPLEMENTARY TABLES

**Supplementary Table 1. The clinical information of 41 patients with benign lung disease for NPAS2 in IHC analysis.**

| | | Cases (n) | Percentage (%) |
|---|---|---|---|
| Gender | Male | 25 | 61.0 |
| | Female | 16 | 39.0 |
| Age | <60 | 27 | 65.9 |
| | ≥60 | 14 | 34.1 |
| Type of diseases | Pulmonary bulla | 24 | 58.6 |
| | Hamartoma | 11 | 26.8 |
| | CCAM | 4 | 9.8 |
| | BPS | 1 | 2.4 |
| | pneumothorax | 1 | 2.4 |

*CCAM, congenital cystic adenomatoid malformation; BPS, bronchopulmonary sequestration.

**Supplementary Table 2. The basic information of 388 patients with LUAD for NPAS2 IHC analysis.**

| | | Cases (n) | Percentage (%) |
|---|---|---|---|
| Gender | Female | 187 | 48.2 |
| | Male | 201 | 51.8 |
| Age | <60 | 178 | 45.9 |
| | ≥60 | 210 | 54.1 |
| Grade | G1 | 33 | 8.5 |
| | G2 | 252 | 64.9 |
| | G3 | 96 | 24.8 |
| | NA | 7 | 1.8 |
| T classification | T1 | 112 | 28.9 |
| | T2 | 205 | 52.8 |
| | T3 | 51 | 13.1 |
| | T4 | 20 | 5.2 |
| N classification | N0 | 217 | 55.9 |
| | N1 | 98 | 25.3 |
| | N2 | 61 | 15.7 |
| | N3 | 4 | 1.0 |
| | NA | 8 | 2.1 |
| M classification | M0 | 366 | 94.3 |
| | M1 | 22 | 5.7 |
| Stage | Stage I | 182 | 46.9 |
| | Stage II | 100 | 25.8 |
| | Stage III | 84 | 21.6 |
| | Stage IV | 22 | 5.7 |

*NA, Not available.

**Supplementary Table 3. The relationship between NPAS2 expression level and clinical pathological characteristics in 388 patients with LUAD.**

| Parameters | Number of cases | NPAS2 IHC expression | | *P* values |
|---|---|---|---|---|
| | | Low (n=204) | High (n=184) | |
| Gender | | | | |
| Female | 187 | 100 | 87 | 0.732 |
| Male | 201 | 104 | 97 | |
| Age | | | | |
| <60 | 178 | 91 | 87 | 0.598 |
| ≥60 | 210 | 113 | 97 | |
| Grade | | | | |
| G1-G2 | 285 | 160 | 125 | **0.035*** |
| G3 | 96 | 42 | 54 | |
| NA | 7 | 2 | 5 | |
| T classification | | | | |
| T1-2 | 317 | 169 | 148 | 0.540 |
| T3-4 | 71 | 35 | 36 | |
| N classification | | | | |
| N0 | 217 | 134 | 83 | **0.0002*** |
| N1-3 | 163 | 69 | 94 | |
| NA | 8 | 1 | 7 | |
| M classification | | | | |
| M0 | 366 | 197 | 169 | **0.045*** |
| M1 | 22 | 7 | 15 | |
| Stage | | | | |
| I-II | 282 | 168 | 114 | **<0.0001*** |
| III-IV | 106 | 36 | 70 | |

*IHC, Immunohistochemistry; NA, Not available.

**Supplementary Table 4. Univariate analysis of NPAS2 expression and clinical pathological characteristics associated with distant metastasis-free survival in 388 patients with LUAD.**

| Parameters | Hazard ratio (95%CI) | *P* values |
|---|---|---|
| Gender (male v.s. female) | 1.379(0.994-1.914) | 0.055 |
| Age (≥60 v.s. <60) | 1.044(0.755-1.444) | 0.795 |
| Grade (G1-G2 v.s. G3) | 1.939(1.516-3.144) | **<0.0001*** |
| T classification (T1-2 v.s. T3-4) | 2.183(1.451-2.967) | **<0.0001*** |
| N classification (N1-3 v.s. N0) | 2.598(1.853-3.641) | **<0.0001*** |
| M classification (M1 v.s. M0) | 4.642(2.812-7.661) | **<0.0001*** |
| Stage (III-IV v.s. I-II) | 4.425(3.188-6.141) | **<0.0001*** |
| NPAS2 expression (high v.s. low) | 2.102(1.507-2.931) | **<0.0001*** |

**Supplementary Table 5. Multivariate analysis of NPAS2 expression and clinical pathological characteristics associated with distant metastasis-free survival in 388 patients with LUAD.**

| Parameters | Hazard ratio (95%CI) | *P* values |
|---|---|---|
| Gender (male v.s. female) | 1.396(0.969-2.013) | 0.074 |
| Grade (G3 v.s. G1-G2) | 1.328(0.900-1.961) | 0.153 |
| T classification (T3-4 v.s. T1-2) | 0.919(0.574-1.472) | 0.726 |
| N classification (N1-3 v.s. N0) | 1.330(0.965-2.045) | 0.194 |
| M classification (M1 v.s. M0) | 2.203(1.112-4.367) | **0.024*** |
| Stage (III-IV v.s. I-II) | 2.901(1.719-4.894) | **<0.0001*** |
| NPAS2 expression (high v.s. low) | 1.509(1.053-2.163) | **0.025*** |
